# Memristors with Nociceptor Characteristics Using Threshold Switching of Pt/HfO_2_/TaOx/TaN Devices

**DOI:** 10.3390/nano12234206

**Published:** 2022-11-26

**Authors:** Minsu Park, Beomki Jeon, Jongmin Park, Sungjun Kim

**Affiliations:** Division of Electronics and Electrical Engineering, Dongguk University, Seoul 04620, Republic of Korea

**Keywords:** RRAM, nociceptor, threshold switching, high-k

## Abstract

As artificial intelligence technology advances, it is necessary to imitate various biological functions to complete more complex tasks. Among them, studies have been reported on the nociceptor, a critical receptor of sensory neurons that can detect harmful stimuli. Although a complex CMOS circuit is required to electrically realize a nociceptor, a memristor with threshold switching characteristics can implement the nociceptor as a single device. Here, we suggest a memristor with a Pt/HfO_2_/TaO_x_/TaN bilayer structure. This device can mimic the characteristics of a nociceptor including the threshold, relaxation, allodynia, and hyperalgesia. Additionally, we contrast different electrical properties according to the thickness of the HfO_2_ layer. Moreover, Pt/HfO_2_/TaO_x_/TaN with a 3 nm thick HfO_2_ layer has a stable endurance of 1000 cycles and controllable threshold switching characteristics. Finally, this study emphasizes the importance of the material selection and fabrication method in the memristor by comparing Pt/HfO_2_/TaO_x_/TaN with Pt/TaO_x_/TaN, which has insufficient performance to be used as a nociceptor.

## 1. Introduction

Among several next-generation memories, resistive random-access memory (RRAM) operates with the resistance state proposed by Chua [1]. Memristors based on RRAM have been extensively studied in various fields such as information processing, material engineering, structural integration, fabrication process, etc. [2,3,4]. These studies focus on filling the gap between storage class memory and traditional memory technologies (flash memory and dynamic random-access memory) and implementing a system for neuromorphic applications and non-Von Neumann computing [5,6,7]. The work to implement neuromorphic computing, which has a human learning algorithm and various thinking skills, has also been actively researched by mimicking brain synapse functions such as spike-time-dependent plasticity (STDP), paired-pulse facilitation (PPF), long-term potentiation (LTP), and long-term depression (LTD) [8,9,10,11,12]. These synaptic characteristics functionally operate based on resistive switching (RS) and are even capable of implementing threshold switching (TS). In particular, TS memristors can react according to external stimuli within a specific range. Due to these characteristics, they can be used in the field of artificial neural networks [13,14,15,16]. For example, biological properties can be conjugated to future research such as intelligent sensory systems and humanoid robots [17]. Additionally, in space research and science, the demand for future space suits that function as human skin has continued to increase among people with acquired neurological disorders [18,19,20]. 

The sensory system needs the ability to detect and efficiently recognize unwanted external pressure, force, mechanical strength, temperature, and danger signals. There are several receptors in the human biometric system, and a nociceptor is one of the most important and essential receptors that generates pain signals to make the body avoid dangers [21,22,23]. The nociceptor operates on an external signal in the following way. When nerve cells are not excited, they show “no adaptation” and “relaxation” behaviors. Conversely, “allodynia” and “hyperalgesia” behaviors are exhibited in the excited state. Here, TS is the key to making an artificial nociceptor. TS does not respond to the signals below a certain value but reacts strongly when the boundary value is exceeded. When the external stimulus beyond the threshold disappears, the nociceptor slowly returns to an unexcited state. In addition, the nociceptors increase the response degree with the signal persistence or injured nociceptors respond to a much lower threshold value. The principle of this system defends the body from external and strong stimuli. For example, it is typical to show a sensitive reaction even if low stimuli are applied to the damaged skin. 

When the nociceptor feels a strong stimulus beyond the threshold, it reaches an excited state and reacts to the stimulus. In an excited state, “allodynia” and “hyperalgesia” are indicators of the degree of stimulus versus the reaction. First, “allodynia” is the act of generating a reaction signal at a value less than the threshold value in an unexcited state, and “hyperalgesia” is the act of generating an excessively strong signal compared to the degree of reaction in an unexcited state above the threshold value. To simulate a nociceptor, a complementary metal–oxide–semiconductor (CMOS) sensor is required but has the disadvantages of increasing power consumption and circuit complexity. Therefore, if a memristor using RRAM with TS characteristics follows nociceptor characteristics, it will be possible to maximize advantages such as a simple MIM structure and a power reduction [13,24,25,26,27].

In this study, Pt/TaO_x_/TaN (PTT) and Pt/HfO_2_/TaO_x_/TaN (PHTT) were investigated. The thickness of HfO2 was varied into 3 nm (PHTT3), 5 nm (PHTT5), and 7 nm (PHTT7) to identify the four critical characteristics (“no adaptation”, “relaxation”, “allodynia”, and “hyperalgesia”) mentioned above. It was confirmed by transmission electron microscope (TEM). HfO_2_, not only well known as a high-k material but also as an emerging material in the field of RRAM, was used, and TaO_x_ with stable switching characteristics was also deposited. When these two overlapped materials were sandwiched by the Pt and TaN, it showed the I–V curve of TS at a low current level. The previous studies focused on implementing self-rectifying characteristics using the TS feature of TaO_x_ and HfO_2_ [28,29]. However, where and how this property can be applied and the effects of the thickness are not described in detail. Thus, we focused on this part to conduct our research. In the case of PTT and PHTT, they were selected as the control group to check how each insulating layer mimicked the nociceptor function. We investigated the I–V curve of PTT and PHTT and the retention characteristic. Furthermore, we proposed a charge-trapping and de-trapping mechanism to explain the TS behaviors. Eventually, we experimentally demonstrated that PHTT has four nociceptive properties. We also anticipate that this device can be applied to artificial intelligence fields such as humanoid robots as nociceptors in the future.

## 2. Experiments

RRAM samples were fabricated using deposition techniques such as sputtering, atomic layer deposition (ALD), and e-beam evaporator. For the Pt/HfO_2_/TaO_x_/TaN device, a TaN (100 nm) with a sheet resistance of 60 Ω/sq was deposited for use as the bottom electrode with DC sputtering (AMAT, ENDURA 5500) on the 20 mm x 20 mm SiO_2_/Si substrate. Then, 20 nm thick TaO_x_ and 3 nm thick HfO_2_ layers were deposited sequentially via pulsed DC reactive sputtering and ALD, respectively. Pulsed DC reactive sputtering of the TaO_x_ film was performed at room temperature. The gas flow rate of Ar and O_2_ were set to 8 sccm and 12 sccm, respectively, the base pressure of 1.6 × 10^−6^ Torr was used, and the deposition pressure was 1 mTorr. The 500 W of DC power (pulsed DC, 50 kHz) was applied. HfO_2_ film with the ALD process was deposited using TEMAHf and H_2_O at the stage temperature of 280 °C using a thermal ALD process (CN1, Atomic premium). One cycle for ALD HfO_2_ is composed of TEMAHF (0.5 s)-purge (35 s)-H_2_O (0.3 s)-purge (35 s). In total, 29 cycles, 49 cycles, and 68 cycles were performed for 3 nm (PHTT3), 5 nm (PHTT5), and 7 nm (PHTT7) of HfO_2_ layers, respectively. Hf and Ta is entirely oxidized for the HfO_2_ and TaO_x_ film. The results of stoichiometry are shown from previous studies in which the device is deposited under the same conditions as PHTT [26]. Finally, Pt (100 nm) was deposited as the top electrode using an e-beam evaporator (ULTEC, FR-EB20). Patterns were engraved utilizing the shadow mask filled with a 100 μm diameter cell. The electrical characteristics of each cell were measured in the DC mode using a Keithley 4200-SCS semiconductor parameter analyzer and in the pulse mode using a 4225-PMU ultrafast module.

## 3. Results and Discussions

**Figure 1:**
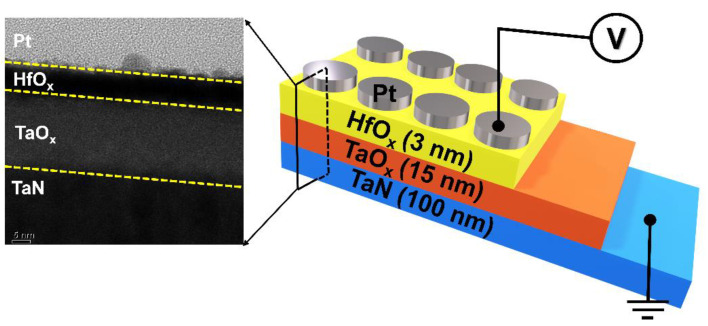
Cross-sectional TEM analysis and schematic of the Pt/HfO_2_/TaO_x_/TaN (PHTT3) device.

Using the TEM analysis, we first identified the structure of the PHTT device as shown in Figure 1. It was confirmed that two insulator layers, TaO_x_ and HfO_2_, were deposited between the top and bottom electrodes by 3 nm and 15 nm, respectively. Other devices, PTT, PHTT5, and PHTT7, were fabricated under the same fabrication condition. To measure the electrical characteristics and response to stimulus, an external bias provoked from the module was applied to the top electrode (Pt), while the bottom electrode (TaN) was grounded.

**Figure 2:**
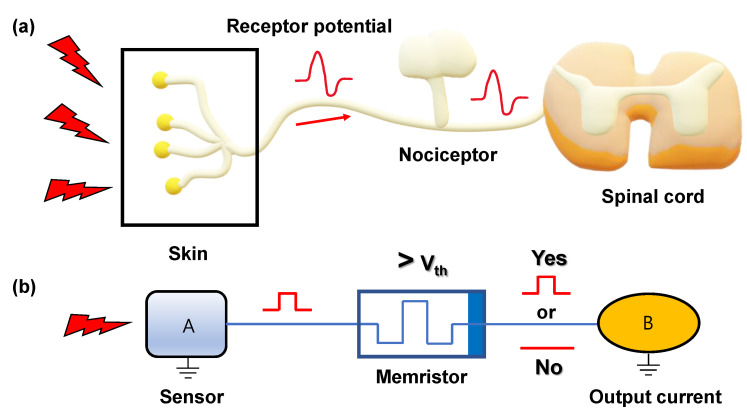
Nociceptor system in a human body and artificial nociceptor circuit using a TS memristor. (**a**) When external stimuli are introduced through the human skin, nociceptor determines the magnitude of the signal above the threshold and creates a potential to send information to the spinal cord. (**b**) Schematic in which an electrical pulse is applied to the sensor. A pulse higher than the threshold value is applied, the memristor is turned on and an output current is generated.

Figure 2 shows a nociceptor system of the human body and TS memristor artificially operating it [30]. In our body, nociceptors are located anywhere at the end of the neuron sensor. The nociceptor recognizes the external stimulus and compares its magnitude with a threshold value to determine whether to generate an electrical signal and send the signal to the brain through the spinal cord. The response to stimuli allows our bodies to defend against dangerous things. If there is no receptor system, we will inevitably be exposed to danger and be defenseless. Similarly, when an electrical pulse is applied to an artificial memristor mimicking a nociceptor, a response occurs according to the pulse amplitude. If the pulse amplitude is greater than the threshold voltage, the memristor is turned on and the output current is generated. This process is described in Figure 2a,b, respectively. The opposite situation operates on a similar principle, but in this case, the memristor is turned off and the output current is not generated. Memristors receiving stimuli above the threshold value begin to be relaxed immediately to correspond to the next stimulus.

**Figure 3:**
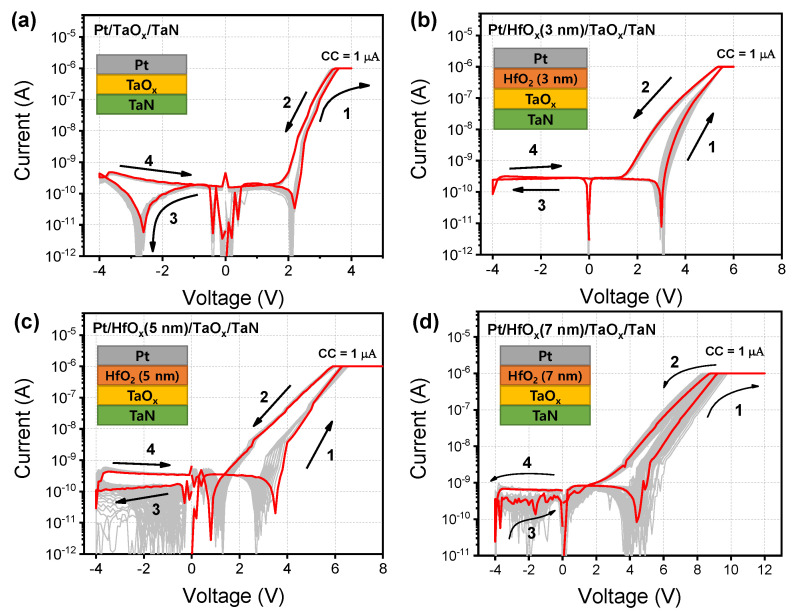
I–V characteristics of four devices: (**a**) PTT, (**b**) PHTT3, (**c**) PHTT5, (**d**) PHTT7. Schemes of the device are shown as the inset.

I–V characteristics of the threshold switching are shown in Figure 3a–d. Each I–V curve shows 100 cycles; the red line shows the 50th curve of positive TS property. A 0.05 V step per sweep and voltage sequence for returning to 0 V though Vset (4/6/8/12 V) and Vreset (−4 V) were given to each device in the condition of current compliance (I_CC_) of about 1 μA. The electroforming process was not shown in the TS characteristics due to the current limitation. Switching direction is marked with the numbers from 1 to 4 in Figure 3a, which shows the I–V curve of PTT and a small V_set_ of approximately 3.7 V. With increasing voltage from 0 V to 4 V, a set process that gradually switched from the high-resistance state (HRS) and low-resistance state (LRS) occurred. Interestingly, after going through a set process, it returned to the HRS even if the device did not experience the reset process. Nevertheless, the reason for the negative voltage was to keep the interval between the DC cycle and cycle constant. PHTT3 in Figure 3b has a V_set_ of about 4.5 V, which is greater than PTT. The V_set_ in Figure 3c,d increases gradually as the thickness of HfO_2_ becomes thicker and the non-uniformity of the I–V curve becomes severe. Among the four devices, PHTT3 shows the most uniform performance, a low V_set_, and the high on–off ratio of ≈21. Thus, we decided to use PHTT3 for the realization of nociceptor function in further study.

**Figure 4:**
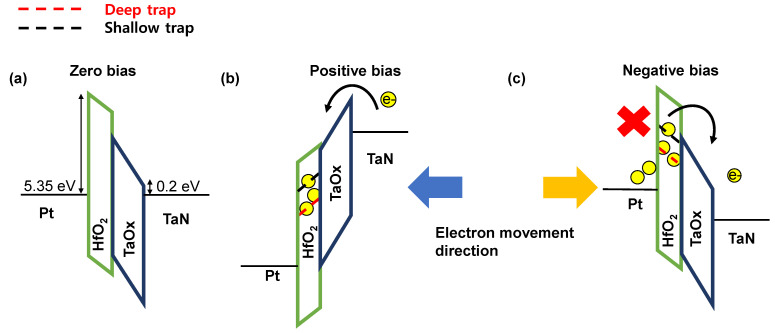
Band diagram schematic of PHTT device. (**a**) Zero bias condition. (**b**) Positive bias condition. (**c**) Negative bias condition.

Figure 4 shows the band diagram schematic of the PHTT device. The conduction band offset of the top and bottom electrode with the insulating layer is shown. As a result of calculating the work function of each material from the zero bias in Figure 4a, the energy barrier between Pt and HfO_2_ is 5.35 eV, and between TaO_x_ and TaN it is 0.2 eV [31,32,33]. The detailed switching process can be described in the following description. The deep and shallow trap sites exist in the insulating layer of HfO_2_. When these traps are not filled with electrons, the device remains in the HRS. Figure 4b illustrates when a positive voltage is applied to the Pt top electrode so that the trap energy level becomes lower than the Fermi level of the TaN bottom electrode [24]. The trap sites in HfO_2_ gradually accumulate with electrons injected from TaN and the PHTT device begins to switch from HRS to LRS. Moreover, depending on the magnitude of I_CC_, it is possible to control the number of electrons being filled in the trap. When the externally applied voltage is removed, the energy band returns to its original state in the zero bias. Contrary to the previous state, the energy level in HfO_2_ is higher than the Fermi level in TaN, resulting in de-trapping in which electrons naturally flow to the TaN bottom electrode. Therefore, PHTT returns from LRS to HRS with the removal of positive voltage. Figure 4c shows the condition of the negative bias; electron de-trapping is further accelerated and blocks the electron injection through the Pt top electrode. Since the trap energy level rises, the de-trapping speed of electrons is also increased. PHTT5 and PHTT7 have thicker HfO_2_ than PHTT3, and a greater number of traps are located in the insulating layer. As confirmed in Figure 3, this may be the reason for the increase in the V_set_ value.

**Figure 5:**
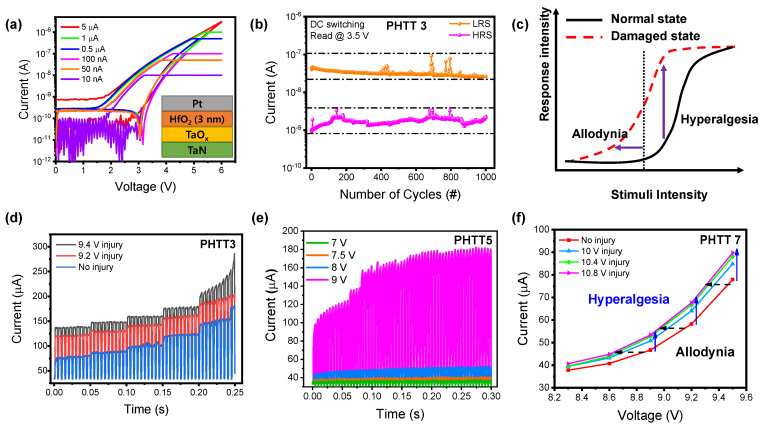
(**a**) TS characteristics of PHTT3 at different I_CC_. (**b**) DC endurance test of PHTT3 went up to ≈10^3^ times. Orange dots and pink dots refer to LRS current and HRS current, respectively. (**c**) Typical stimulus versus response relation in the nociceptor in the normal and damaged states. The arrows represent “allodynia” and “hyperalgesia” characteristics. (**d**) Current response according to the voltage in the PHTT3 with and without an injury. (**e**) Current responsivity according to different voltage at PHTT5. (**f**) Experimentally measured current of the PHTT7 before and after the pulse injury, showing the appearance of “allodynia” and “hyperalgesia” characteristics.

Figure 5a shows different TS behaviors at various I_CC_ values. The PHTT3 device shows a large range of TS characteristics depending on I_CC_ from a minimum of 10 nA to a maximum of 5 µA, showing that our device not only operated at a low I_CC_ but was also driven at a high I_CC_. The endurance test with 1000 cycles was checked as shown in Figure 5b. Endurance was measured by applying a DC voltage of 6 V (SET), reading through a DC voltage of 3.5 V, applying a DC voltage of −4 V (RESET), and reading at 3.5 V. The resistance values were extracted from the read voltage of 3.5 V, and stable endurance was achieved compared to the previously reported work [28]. Next, we experimentally confirmed the characteristics of the nociceptors, called “allodynia” and “hyperalgesia”. Figure 5c schematically indicates the signal responses of the nociceptors in damaged and normal states, and “allodynia” and “hyperalgesia” behaviors are separated by the threshold value. After the injury, the nociceptor must show the enhanced response at a reduced threshold as presented by vertical and horizontal arrows. Figure 5d expresses the current response under the different voltages (pulse amplitude: 4.5 V~8.5 V/step: 1 V). The width of the pulse was fixed at 0.3 ms. First, a current of 75 µA flowed at a pulse amplitude of 4.5 V without damage. As the pulse amplitude increased, the output current reached a maximum of 150 µA. Second, the red line was damaged with a strong pulse of 9.2 V before pulse measurement, and then the current response was measured. The current raised from 120 µA to 170 µA as the voltage increased. Lastly, because of the strong injury (9.4 V pulse), the current increased rapidly by 10 pulses of 8.5 V amplitude. Similar results were derived through measurements in PHTT5 and PHTT7. As shown in Figure 5e, the voltage was applied from 7 V to 9 V to show the current responses. Figure 5f shows the behavior of “hyperalgesia” and “allodynia”; only the magnitude of the injured voltage is different from Figure 5d. The thickness of the insulating layer was thicker than that of the PHTT3, so a larger voltage was applied to give damage. Through Figure 5d–f, we confirmed that the device reduces the threshold of their external stimuli, generating a current response in previous harmless stimuli (called “allodynia”) and increasing their response to normally innocuous stimuli (called “hyperalgesia”). From these results, it can be suggested that the PHTT device is sufficient to use as the nociceptor characteristics.

**Figure 6:**
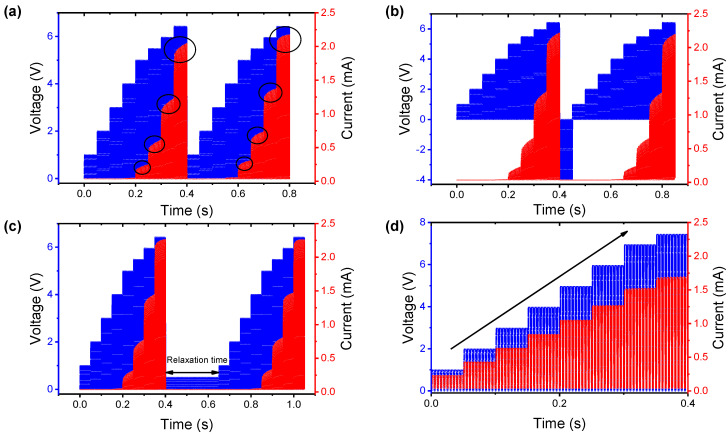
Current response with stair-shaped voltage in PTT. (**a**) Different amplitudes (1 V, 2 V, 3 V, 4 V, 5 V, 5.5 V, 6 V, and 6.5 V) are applied with a pulse width of 3 ms. (**b**) Negative voltage (−4 V) is applied between stepped pulses. (**c**) Sufficiently long relaxation time of 160 µs and 0.5 V amplitude is applied between stepped pulses so as to not affect the current response. (**d**) Voltage (1 V, 2 V, 3 V, 4 V, 5 V, 6 V, 7 V, and 7.5 V) is applied in PTT that does not function as a nociceptor memristor.

PTT also conducted experiments to check the current response by applying pulses to see if they show the characteristics of memristor nociceptors such as PHTT. Additionally, we experimented with two repetitive pulses rather than a single set of pulses. In Figure 6a, stair-shaped voltages (1 V, 2 V, 3 V, 4 V, 5 V, 5.5 V, 6 V, and 6.5 V) were applied, and after 0.4 s, the same pulses were applied immediately without a relaxation time. PTT did not respond to 4 V with a pulse width of 3 ms, and then a current flowed from a voltage higher than 4 V. The current is slightly increased compared with the current flowing in the first pulse (black circle mark). This corresponds to the “allodynia” and “hyperalgesia” characteristics of the nociceptor, respectively. Here we went a step further, adding two variable factors to prevent the current increase. As shown in Figure 6b, negative voltage (−4 V) was inserted between pulses, and instead of a negative voltage, a sufficiently long time of 160 µs was applied in a way that did not affect the device, as shown in Figure 6c. This suggests that in PTT, “no adaptation” and “relaxation” depend on the negative voltage or the relaxation time. Finally, we checked the current response according to the voltage when the nociceptor function was not fully broken. Before pulse measurement, a breakdown state, in which the role of the insulating layer disappeared, was demonstrated by applying a high voltage. After that, Figure 6d shows the characteristics of the current flowing in response to each voltage. Thus, PTT in the breakdown state is not appropriate for the nociceptor memristor at all. 

## 4. Conclusions

We successfully fabricated an artificial nociceptor of PTT and PHTT devices. Four properties were experimentally demonstrated to show “no adaptation”, “relaxation”, “allodynia”, and “hyperalgesia”. In addition, we analyzed the functional operation of the nociceptors using the I–V curve and pulse voltage of PTT, PHTT3, PHTT5, and PHTT7 devices. In addition, the experiment that varied the thickness confirmed that PHTT3 could realize the most optimal artificial nociceptor concept in various aspects such as V_set_, DC switching stability, and current response by external pulse voltage. In accordance with the previous results, it can be strongly argued that the materials and structures using PHTT are suitable for realizing artificial nociceptors in the future.

## Data Availability

Not applicable.
